# Quantifying carboxymethyl lysine and carboxyethyl lysine in human plasma: clinical insights into aging research using liquid chromatography-tandem mass spectrometry

**DOI:** 10.1186/s12896-024-00838-5

**Published:** 2024-03-07

**Authors:** Daguang Wang, Junshan Wang, Xinghong Liu, Kehe Du, Hongjun Liu, Xiaofeng Yang, Tianyi Liu, Qian Liu, Meng Wang, Jian Guo

**Affiliations:** 1 Clinical Laboratory, Aviation General Hospital, Beijing, 100000 China; 2 Iphase Pharma Laboratory, Iphase Pharma Services (Think Tank Research Center for Health Development Laboratory), Beijing, 100000 China; 3 Beijing Hospital Laboratory, National Center for Gerontology, No.1 of Dahua Road, Dongcheng District, Beijing, 100000 China

**Keywords:** CEL, CML, Human aging, Human plasma, LC-MS/MS

## Abstract

**Objective:**

The objective of this study was to establish a methodology for determining carboxymethyl lysine (CML) and carboxyethyl lysine (CEL) concentrations in human plasma using liquid chromatography-tandem mass spectrometry (LC-MS/MS). The test results were also used for clinical aging research.

**Methods:**

Human plasma samples were incubated with aqueous perfluorovaleric acid (NFPA), succeeded by precipitation utilizing trichloroacetic acid, hydrolysis facilitated by hydrochloric acid, nitrogen drying, and ultimate re-dissolution utilizing NFPA, followed by filtration. Cotinine-D_3_ was added as an internal standard. The separation was performed on an Agela Venusil ASB C_18_ column (50 mm × 4.6 mm, 5 μm) with a 5 mmol/L NFPA and acetonitrile/water of 60:40 (v/v) containing 0.15% formic acid. The multiple reaction monitoring mode was used for detecting CML, CEL, and cotinine-D_3_, with ion pairs m/z 205.2 > 84.1 (for quantitative) and m/z 205.2 > m/z 130.0 for CML, m/z 219.1 > 84.1 (for quantitative) and m/z 219.1 > m/z 130.1 for CEL, and m/z 180.1 > 80.1 for cotinine-D_3_, respectively.

**Results:**

The separation of CML and CEL was accomplished within a total analysis time of 6 minutes. The retention times of CML, CEL, and cotinine-D_3_ were 3.43 minutes, 3.46 minutes, and 4.50 minutes, respectively. The assay exhibited linearity in the concentration range of 0.025–1.500 μmol/L, with a lower limit of quantification of 0.025 μmol/L for both compounds. The relative standard deviations of intra-day and inter-day were both below 9%, and the relative errors were both within the range of ±4%. The average recoveries were 94.24% for CML and 97.89% for CEL.

**Conclusion:**

The results indicate that the developed methodology is fast, highly sensitive, highly specific, reproducible, and suitable for the rapid detection of CML and CEL in clinical human plasma samples. The outcomes of the clinical research project on aging underscored the important indicative significance of these two indicators for research on human aging.

## Introduction

The term Advanced Glycation End-products (AGEs) refers to a category of stable end-products resulting from a series of reactions such as condensation, rearrangement, cleavage, and oxidative modification that occur between the carbonyl groups of reducing sugars and the free amino groups of substances such as proteins, lipids, amino acids, or nucleic acids [1]. Medical studies have shown that AGEs are generated and accumulate in serum and tissues as individuals age. They are involved in the onset and progression of a variety of diseases and aging, including the development of chronic complications associated with diabetes, atherosclerosis, uremia, Alzheimer’s disease, and cataracts [2–4].

In order to comprehensively assess the impact of AGEs on human health, it is imperative to employ accurate and reliable methods for the measurement of appropriate biomarkers. CML, CEL, pyrraline, and pentosidine are among the over 20 AGEs that have been identified [5]. CEL and CML, in particular, are closely associated with aging as well as diseases such as diabetic complications and renal failure [6–9]. The development of an efficient and accurate quantitative method for the determination of CML and CEL assumes much significance for advancing further clinical research on the correlation between CML, CEL, and the aging process, as well as for assessing overall human health status.

Commonly employed methods for the determination of CML and CEL in biological samples include HPLC-fluorescence detection (FLD) with pre-column derivatization using ortho-phtaldialdehyde (OPA) [10] or 6-aminoquinolyl-N-hydroxysuccinimidyl carbamate (AQC) [11], gas chromatography-mass spectrometry (GC-MS) method by analyzing N(O,S)-ethoxycarbonyl ethyl esters of amino acid derivatized by ethylchloroformate [12], enzyme-linked immunochemical quantification of CML with a specific antibody, [9, 13] and the HPLC with stable-isotope-dilution tandem mass spectrometry method [14–16], among others. Among these, HPLC-MS/MS has obvious advantages because the use of multiple reaction monitoring (MRM) mode improves sensitivity, reduces the coefficient of variability, eliminates the problems of derivatization, and allows for the simultaneous analysis of multiple analytes.

In this experiment, an efficient, sensitive, accurate, and specific technique was developed utilizing LC-MS/MS for the accurate determination of CML and CEL in human plasma.

## Materials and methods

### Samples, standard materials, and reagents

#### Standard materials and samples

CML (95% purity) and CEL (96% purity) were purchased from TMRM (China). Cotinine-D_3_ (99% purity) was bought from Cerilliant (USA). A total of 1196 clinical plasma samples were provided by the Institute of Geriatrics, Beijing Hospital, China.

#### Reagents

Methanol, acetonitrile, and formic acid, all of chromatography grade, were sourced from Burdick & Jackson (USA). The ultra-pure water that was utilized in the experiments was prepared using the Milli-Q water purification system. The NFPA used was of analytical reagent quality and obtained from Macklin (China). Trichloroacetic acid (TCA) and hydrochloric acid, both analytical reagents, were purchased from Sinopharm Chemical Reagent Co., Ltd. (China). Additionally, 4% bovine serum albumin prepared with saline was utilized.

### Detection methods

#### LC-MS/MS

A triple quadruple mass spectrometer (MS), API 4000 (Applied Biosystems, USA), coupled to a LC system manufactured by Shimadzu (Japan) was utilized. The MS was operated in a positive ion mode using electrospray ionization (ESI) on a turbo-ion spray source. Multiple reaction monitoring (MRM) was used for the MS analyses. Ion pairs selected for analysis were m/z 205.2 > m/z 84.1 (for quantitative) and m/z 205.2 → m/z 130.0 for CML, m/z 219.1 > m/z 84.1 (for quantitative) and m/z 219.1 > m/z 130.1 for CEL, and m/z 180.1 > m/z 80.1 for cotinine-D_3_, respectively. The temperature of the auxiliary gas was set to 550 °C, the ion spray voltage was 5500 V, and the de-clustering potentials were 56 V, 56 V, 25 V, 25 V, and 64 V, respectively. Collision energies (CE) were set at 17 eV, 27 eV, 27 eV, 18 eV, and 30 eV, respectively. Nitrogen was used as the curtain, turbo-ion spray, collision, and nebulizer gas in the system.

Analyst 1.4.2 software was used for all data acquisition and processing in this study. The HPLC separation was performed using a chromatographic column (Lightn AQ 4.6 × 50 mm, 5-μm particles; Venusil ASB C_18_, Agela, USA). The composition of the solution was as follows: eluent A, 5 mmol/L NFPA, and eluent B, acetonitrile/water (v/v) = 6:4 (containing 0.15% formic acid). Eluent B (5%) underwent a linear gradient increase from 5 to 25% over 0.50 minutes, followed by a linear gradient increase from 25 to 60% over 3.51 minutes, and from 60 to 98% over 0.99 minutes. Subsequently, Eluent B returned to 5% over 0.01 minutes and remained at 5% for 1 minute. The flow rate was 1.00 mL/min, and the injection volume was 10 μL.

#### Standard curves and quality control (QC) samples

The composite standard curve samples for CML and CEL consisted of standard curve samples spiked with incremental concentrations of working standard solution of 0.250, 0.500, 1.000, 3.000, 4.500, 7.000, 12.000, and 15.000 μmol/L. These samples were prepared with 4% bovine serum albumin as the matrix and were tailored for specific requirements. The mixed QC samples for CML and CEL comprised QC samples spiked with serial concentrations of QC working standard solution of 1.500, 4.500, and 10.000 μmol/L formulated with 4% bovine serum albumin as the matrix. These were prepared as required.

#### Plasma sample processing

The analytical procedure involved pipetting 50 μL of the plasma sample to be analyzed or the blank sample into a 10 mL centrifuge tube with a screw cap. Next, 100 μL of deionized water and 500 μL of 5 mmol/L NFPA were added, and the mixture was incubated at room temperature for 2 hours. After that, 2 mL of TCA with a concentration of 200 g/L was added, and the sample was centrifuged at 2000 g for 10 minutes. The supernatant was removed, and the remaining solution was washed with 1 mL of TCA with a concentration of 100 g/L. The supernatant was washed by adding 1 mL of 100 g/L TCA, centrifuged at 2000 g for 10 minutes, and removed. The remaining solution was added to 500 μL of a 6 mol/L hydrochloric acid solution and subjected to hydrolysis in an oven at 110 °C for 20 hours. The hydrolyzed product was dried using nitrogen gas in a water bath at 80 °C, then added to 300 μL of NFPA (5 mmol/L). The remaining solution was drawn using a 1 mL syringe and filtered through a 0.22 μm membrane. A 200-μL aliquot of the membrane-filtered solution was taken and mixed well with 20 μL of Cotinine-D_3_ (500 ng/mL, prepared with 50% acetonitrile-water). Finally, the resulting mixture was subjected to LC-MS/MS analysis.

#### Data collection and processing

The chromatograms of CML, CEL, and Cotinine-D_3_ were integrated using the Analyst version 1.4.2 software. Linear regression was performed using the area ratio of CML or CEL to Cotinine-D_3_ as the vertical coordinate and the corresponding concentration of CML or CEL as the horizontal coordinate, with a weighting coefficient of 1/x^2^. The concentration of CML or CEL in the unknown samples was calculated using the following formula:

$$x=\frac{y-b}{a}$$

Wherein, y represents the ratio of the peak area of CML or CEL to that of its internal standard Cotinine-D_3_; b is the intercept of the standard curve; and a is the slope of the standard curve.

## Results

### Methodological validation

#### Standard curve and lower limit of quantification (LLOQ)

**Fig. 1:**
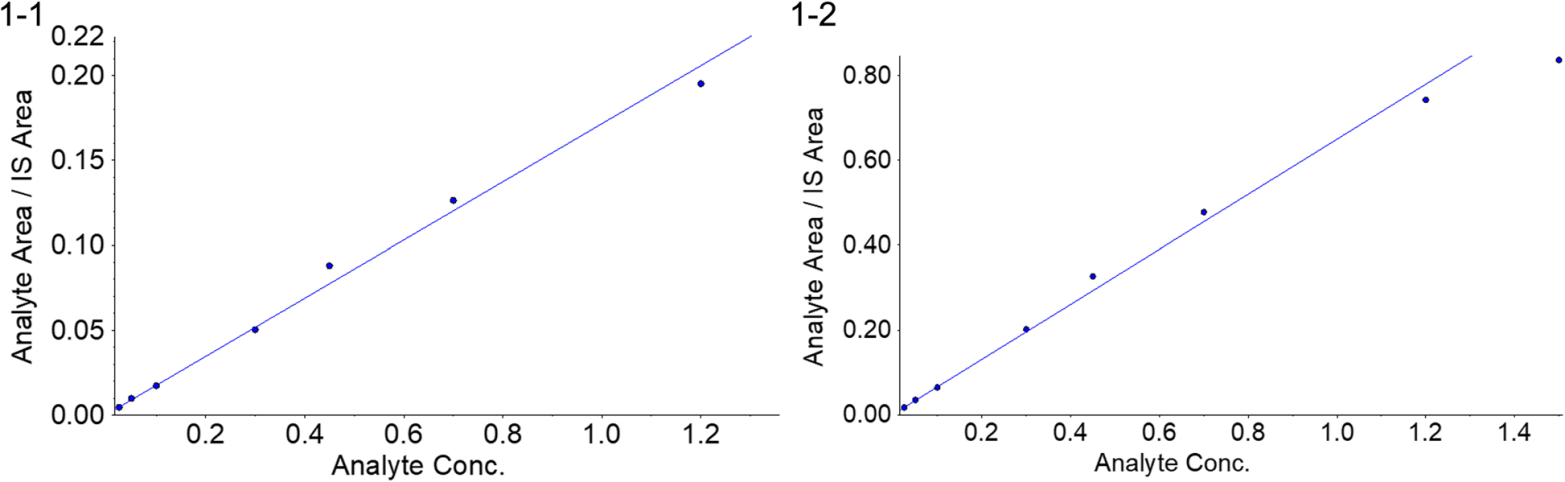
1-1: Standard curve of CML in human plasma. 1-2: Standard curve of CEL in human plasma

**Fig. 2:**
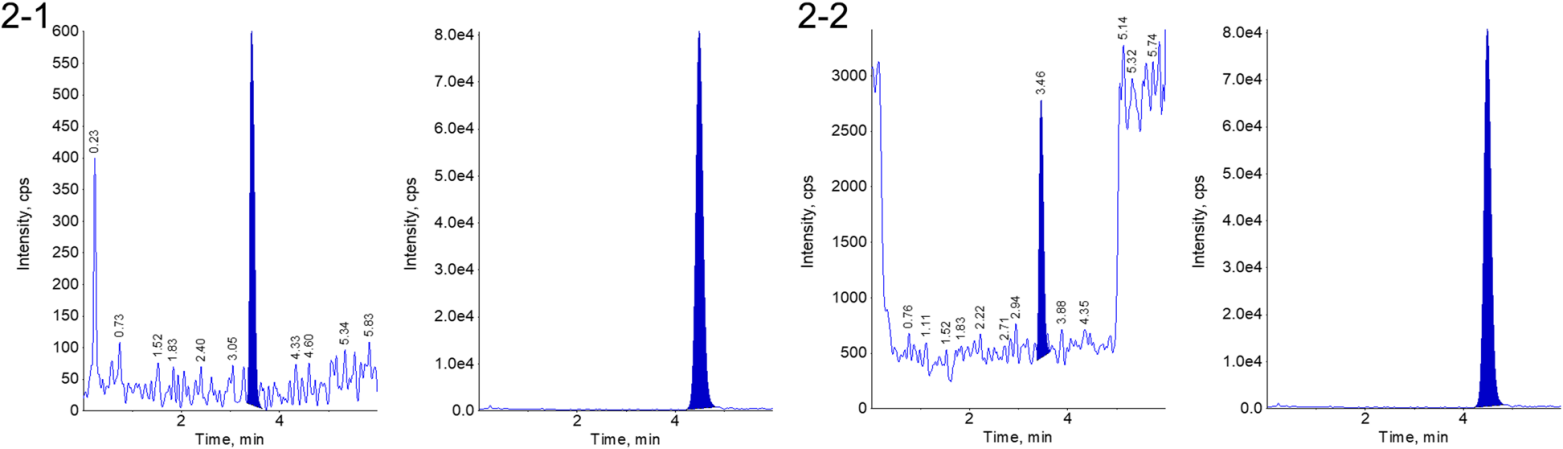
2-1: LLOQ of CML in human plasma. 2-2: LLOQ of CEL in human plasma

For determination of the standard curve and lower limit of quantification (LLOQ), 20 μL of each standard sample with varying concentrations was combined with 20 μL of Cotinine-D_3_ (500 ng/mL, prepared with 50% acetonitrile water) in a 1.5 mL microcentrifuge tube and then added 180 μL of a 5-mmol/L NFPA aqueous solution. This was mixed well and then analyzed using LC-MS/MS. Each analytical batch incorporated one standard curve, and five consecutive analytical batches were analyzed. The results are shown in Table 1, and the typical working curves of CML and CEL are shown in Fig. 1-1 (correlation coefficient R^2^ = 0.9886) and Fig. 1-2 (correlation coefficient R^2^ = 0.9920), respectively.

**Table 1:** Standard curve results of the determination of CML and CEL in human plasma

Lot number	CML standard curve series concentration (μmol/L)											
	0.025	0.050	0.100	0.300	0.450	0.700	1.200	1.500	LLOQ	Ranges	curve equations	linearity
1	0.025	0.048	0.104	0.316	0.482	0.702	1.060	1.478	0.025	0.025-1.500	y = 0.140x-0.000076	0.9954
2	0.024	0.056	0.097	0.291	0.475	0.738	1.087	1.461	0.024	0.025-1.500	y = 0.137x-0.001250	0.9928
3	0.026	0.043	0.115	0.270	0.497	0.719	1.114	1.502	0.026	0.025-1.500	y = 0.097x-0.000530	0.9862
4	0.025	0.052	0.089	0.257	0.486	0.725	1.277	1.531	0.025	0.025-1.500	y = 0.081x-0.000298	0.9908
5	0.026	0.045	0.099	0.285	0.501	0.775	1.164	1.382	0.026	0.025-1.500	y = 0.180x-0.000395	0.9910
Mean	0.025	0.049	0.101	0.284	0.488	0.732	1.140	1.471	0.025			
SD	0.1	0.1	0.1	0.1	0.1	0.1	0.1	0.1	0.1			
Relative standard deviation (RSD) (%)	3.4	10.8	9.6	7.9	2.2	3.8	7.5	3.9	3.4			
Relative error (RE) (%)	0.8	−2.4	0.8	−5.4	8.5	4.5	−5.0	−1.9	0.8			
N	5	5	5	5	5	5	5	5	5			
Lot number	CEL standard curve series concentration (μmol/L)											
	0.025	0.050	0.100	0.300	0.450	0.700	1.200	1.500	LLOQ	Ranges	curve equations	linearity
1	0.025	0.048	0.099	0.315	0.506	0.724	1.064	1.414	0.025	0.025-1.500	y = 0.624x-0.000541	0.9928
2	0.024	0.054	0.100	0.281	0.496	0.721	1.116	1.442	0.024	0.025-1.500	y = 0.570x-0.002710	0.9946
3	0.026	0.047	0.090	0.292	0.502	0.727	1.048	1.669	0.026	0.025-1.500	y = 0.354x-0.001160	0.9880
4	0.026	0.048	0.091	0.277	0.486	0.728	1.246	1.496	0.025	0.025-1.500	y = 0.316x-0.001310	0.9936
5	0.025	0.049	0.098	0.289	0.516	0.754	1.118	1.371	0.026	0.025-1.500	y = 0.704x-0.001650	0.9918
Mean	0.025	0.049	0.096	0.291	0.501	0.731	1.118	1.478	0.025			
SD	0.1	0.1	0.1	0.1	0.1	0.1	0.1	0.2	0.1			
Relative standard deviation (RSD) (%)	3.4	5.7	5.0	5.1	2.3	1.9	7.0	7.9	3.4			
Relative error (RE) (%)	0.8	−1.6	−4.4	−3.1	11.4	4.4	−6.8	−1.4	0.8			
N	5	5	5	5	5	5	5	5	5

Figure 2-1 and 2-2 illustrate the LLOQ chromatograms of CML and CEL, respectively. The signal-to-noise ratio, as calculated by the software, was greater than 10, and the LLOQ of CML and CEL were both 0.025 μmol/L, thereby meeting the established criteria for quantitative detection.

#### Accuracy and precision

The QC samples were processed with low, moderate, and high concentrations of CML and CEL as per the procedure outlined in Section Standard curve and lower limit of quantification (LLOQ). The concentrations of QC samples were measured with the corresponding working curve of the analytical batch, and the accuracy and precision of the method were calculated based on the measured results of the QC samples. The results are shown in Table 2. The relative errors (REs) were all within ±15%, and the intra-batch relative standard deviation (RSD) and inter-batch RSD were both less than 15%, satisfying the stipulated requirements for accuracy and precision.

**Table 2:** Accuracy and precision of the LC-MS/MS method for the determination of CML and CEL in human plasma

	Theoretical concentration (μmol/L)	Measured concentration (μmol/L)	RE (%)	Intra-batch RSD (%)	Inter-batch RSD (%)
CML	0.15	0.149 ± 0.013	−0.9	6.6	4.1
	0.45	0.440 ± 0.047	−2.3	6.3	5.1
	1.00	0.964 ± 0.110	−3.6	3.6	6.5
CEL	0.15	0.146 ± 0.013	−2.5	9.0	4.2
	0.45	0.465 ± 0.030	3.3	4.3	5.6
	1.00	0.961 ± 0.125	−3.9	2.9	6.5

#### Extraction recoveries

The QC samples of CML and CEL with low, moderate, and high concentrations were processed as per the procedure detailed in Section Standard curve and lower limit of quantification (LLOQ). Three parallel samples were set up for each sample, which were then analyzed using LC-MS/MS to obtain the peak areas of CML and CEL. A volume of 50 μL from the blank matrix (comprising 4% bovine serum albumin) was dispensed and subjected to the protocol outlined in Section Plasma sample processing, designated “Plasma Sample Processing.” It was blown dry with nitrogen, and then 10 μL of a mixture of CML and CEL with the corresponding concentration were added to 290 μL of NFPA (5 mmol/L).

Following the remaining steps specified in the section on “Plasma Sample Processing”, three parallel samples were set up for each sample and analyzed using LC-MS/MS to obtain the peak areas of CML and CEL. At each concentration, the average peak area of CML and CEL obtained by the previous treatment method was divided by the average peak area of CML and CEL obtained by the latter treatment method to calculate the extraction recovery rates. The extraction recoveries of CML were 89.16, 92.91, and 100.64% for low, medium, and high concentration plasma samples, respectively, with an average extraction recovery of 94.24%, while it was 97.63, 97.48, and 98.57% for CEL, respectively, with an average extraction recovery of 97.89%. The results are shown in Table 3.

**Table 3:** Extraction recovery rate of the LC-MS/MS method for the determination of CML in human plasma

Lot number	Experimental data about CML recovery rate (peak area)						Experimental data about CEL recovery rate (peak area)					
	0.150 μmol/L		0.450 μmol/L		1.000 μmol/L		0.150 μmol/L		0.450 μmol/L		1.000 μmol/L	
	A	B	A	B	A	B	A	B	A	B	A	B
1	43,077	44,283	129,667	133,188	277,543	276,647	190,615	195,451	571,266	574,928	1,182,238	1,192,645
	37,761	43,120	118,137	128,090	258,082	262,827	181,802	190,568	531,581	547,983	1,143,725	1,146,286
	37,892	45,765	117,655	132,063	277,598	268,551	189,690	189,743	548,393	570,955	1,169,465	1,207,275
Mean	39,576.67	44,389.33	121,819.67	131,113.67	271,074.33	269,341.67	187,369.00	191,920.67	550,413.33	564,622.00	1,165,142.67	1,182,068.67
SD	3032.09	1325.70	6800.26	2678.31	11,251.72	6943.84	4843.30	3085.06	19,919.49	14,546.08	19,616.95	31,840.36
RSD/%	8%	3%	6%	2%	4%	3%	3%	2%	4%	3%	2%	3%
%A = (A /B)*100%	89.16		92.91		100.64		97.63		97.48		98.57	
mean %A	94.24						97.89					

“A” indicates the method before processing; “B” indicates the method after processing

#### Specificity

**Fig. 3:**
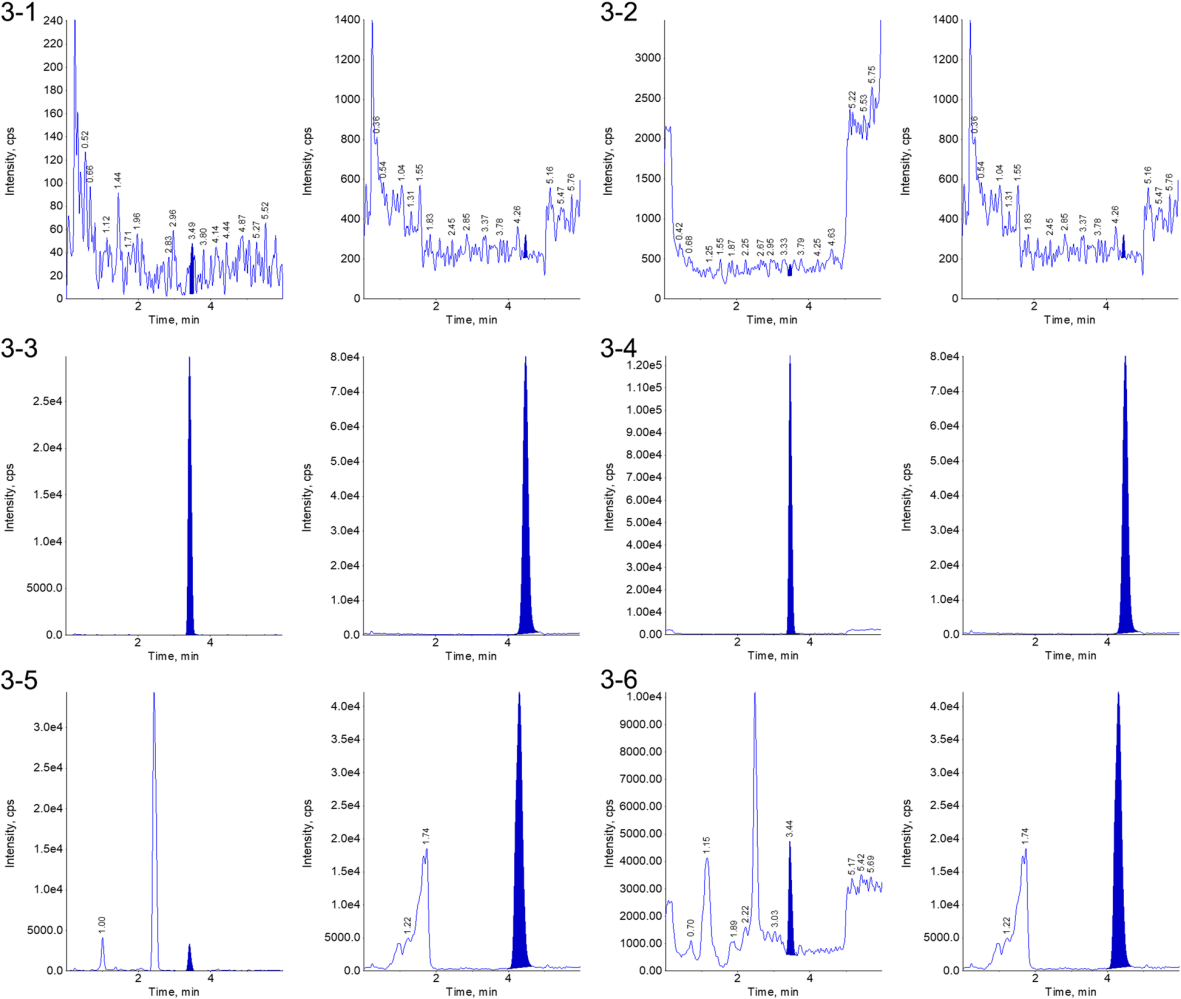
3-1: Chromatogram of CML in 4% bovine serum albumin. 3-2: Chromatogram of CEL in 4% bovine serum albumin. 3-3: Chromatogram of CML and IS in 4% bovine serum albumin. 3-4: Chromatogram of CEL and IS in 4% bovine serum albumin. 3-5: Chromatogram of CML and IS in 4% bovine serum albumin. 3-6: Chromatogram of CEL and IS in 4% bovine serum albumin

**Fig. 4:**
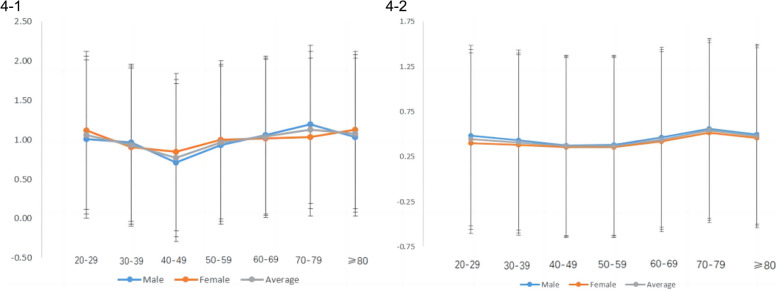
4-1: Changes in CML values with increasing age. 4-2: Changes in CEL values with increasing age

For specificity evaluation, 50 μL of 4% bovine serum albumin was employed, and an equivalent volume of 50% acetonitrile water was substituted for the internal standard solution. The methodology outlined in the “Plasma Sample Processing” section was then adhered to for the generation of MRM chromatograms pertaining to the blank samples (refer to Fig. 3-1 and 3-2). CML, CEL, and cotinine-D3 internal standards were introduced into 4% bovine serum albumin samples. Subsequently, the procedures were conducted in accordance with the protocol delineated in the section titled “Plasma Sample Processing,” resulting in the acquisition of MRM chromatograms for the spiked samples (Fig. 3-3 and 3-4). Actual plasma samples were subjected to the procedures described in the “Plasma Sample Processing” section to obtain the MRM chromatograms of the actual samples (Fig. 3-5 and 3-6). The results showed that the endogenous substances in 4% bovine serum albumin did not interfere with the determination of CML, CEL, or the internal standard. Hence, the method exhibited good specificity.

### Use of the method in clinical samples

The clinical protocols used in this study were approved by the ethics committee of the institution, and the principles outlined in the Declaration of Helsinki were complied with. Informed consent was obtained from all study participants. A comprehensive analysis was conducted on a total of 1196 clinical plasma samples, which were provided by the Institute of Geriatrics at Beijing Hospital, China. The primary objective of this study was to investigate the variations in CML and CEL levels in relation to advancing age.

#### Comparison of CML and CEL between different age groups

The samples were categorized into the following four groups based on the age of the study participants: the youth group: ≤ 44 years old; the middle-aged group: 45–59 years old; the elderly group: 60–74 years old; and the advanced age group: ≥ 75 years old. Analysis of variance (ANOVA) between groups was performed to examine the differences in CML and CEL between these age-based groups. As there were some differences in the indicators for male and female participants, separate ANOVA analyses were done for men and women. Results are shown in Table 4.

**Table 4:** Analysis of variance (ANOVA) results

Indicator	Youth group	Middle-aged group	Elderly group	Advanced age group	*F*-value	*P*-value
CML (μmol/L) (male)	0.96 ± 0.41 (120)	0.85 ± 0.44 (166)	1.10 ± 0.59 (213)	1.11 ± 0.81 (115)	7.38	0
CML (μmol/L) (female)	0.98 ± 0.39 (134)	0.96 ± 0.47 (138)	1.01 ± 0.5 (201)	1.11 ± 0.53 (106)	2.47	0.06*
CEL (μmol/L) (male)	0.46 ± 0.20 (120)	0.37 ± 0.20 (166)	0.50 ± 0.28 (213)	0.51 ± 0.32 (115)	9.75	0
CEL (μmol/L) (female)	0.39 ± 0.19 (134)	0.34 ± 0.18 (138)	0.44 ± 0.27 (201)	0.50 ± 0.30 (106)	10.22	0

A statistically significant difference is indicated when *P* ≤ 0.05, * indicates no significant difference

The results indicate significant differences in CML and CEL between the various age-based groups in the male cohort. In the female cohort, there was a significant difference between the different groups only in CEL levels.

#### Correlation analyses of CML and CEL with age

Pearson’s correlation coefficients for CML and CEL in relation to age were computed separately for each gender, and the corresponding findings are presented in Table 5.

**Table 5:** Results of correlation analysis of the indicators and age

Indicator (male)	Correlation coefficient	*P* value	Indicator (female)	Correlation coefficient	*P* value
CML	0.135	0.001	CML	0.081	0.053
CEL	0.122	0.002	CEL	0.170	0

For the correlation between results and aging, male correlation is higher than female correlation for CML, female correlation is higher than male correlation for CEL.

#### Analyses of the degree of change in CML and CEL with aging

The samples were stratified based on 10-year age intervals. Given the limited number of individuals aged over 90 years, they were aggregated with the age group of 80 years and above. Consequently, the ultimate analysis comprised a total of seven distinct age groups. The mean values and variances of the indicators were computed for each age group. Subsequently, the curves depicting the mean changes for each gender were graphically represented and overlaid on the same chart. Notably, the results showed that the change rate of CML and CEL test results with age was greater in male than in female. (Fig. 4-1 and 4-2).

#### Quantification of the degree of change in CML and CEL

In order to quantify the trends in the alterations of CML and CEL with age, percentage increases (or decreases) for each biomarker were computed over 10-year intervals. Notably, it was observed that the average change in CML and CEL levels every 10 years remained inconspicuous below the age of 40, generally registering below 5%. A distinct upward trend became evident after the age of 40. Consequently, for the age group exceeding 40 years, the average magnitudes of change in the two indicators were recalculated every 10 years. The outcomes indicated a noteworthy increase in the respective degrees of change for CML and CEL, as elucidated in Table 6.

**Table 6:** Results of analysis of degree of change in indicators with age

Indicator	Average degree of change per decade after age 40 (%)		
	Total	Male	Female
CML	Increased by 9.06	Increased by 10.89	Increased by 7.39
CEL	Increased by 8.00	Increased by 8.56	Increased by 7.76

This preliminary determination of the respective degrees of change of CML and CEL with age has considerable significance in guiding the development of a new laboratory assessment system for human health status. Such a system can track the physical status of the group, improve the human health status monitoring network, and promote the establishment of a system for the analysis and management of dynamic data pertaining to monitoring human health.

## Discussion

CML and CEL are two lysine derivatives with potential clinical significance in research on aging. Levels of CML, a metabolite associated with aging, tend to be generally low in the elderly. Studies have shown that CML expression is associated with a variety of age-related diseases and physiological processes, such as cardiovascular disease [17], chronic inflammation [18], and decreased brain function [19]. As a biomarker, CML may be useful in predicting disease risk and assessing health status in the elderly.

CEL is one of the AGEs that is formed by the non-enzymatic glycosylation of glucose or other reducing sugars with lysines in proteins. Studies have shown that CEL levels can be used to assess the extent of the glycosylation reaction and the degree of glycosylation of proteins [20]. Elevated levels of CEL have been linked to a variety of diseases, particularly diabetes and vascular disease. The accumulation of CEL due to hyperglycemia and increased glycosylation reactions can trigger inflammatory responses and oxidative stress, thereby promoting disease progression. Reports on the impact of CEL on cellular genome damage indicate its potential detrimental effects on the genomic integrity of cells [21]. Cellular experiments have shown that the presence of CEL can lead to DNA damage and chromosomal aberrations [21], which in turn increase the risk of cellular genotoxicity and mutation, among others.

In this study, we developed an analytical method using LC-MS/MS for the quantification of CML and CEL in human plasma samples. The method exhibited good selectivity, high sensitivity, and high throughput. Optimized chromatographic conditions significantly reduced the detection time, with only 6 minutes required for the simultaneous detection of both components in each sample. This contributed to improving working efficiency. This method requires only a small volume of plasma (50 μL). The method achieved an LLOQ of 0.025 μmol/L, significantly improving the detection rate of CML and CEL in clinical plasma samples. Furthermore, a comprehensive methodological validation of the detection method was conducted to ensure its accuracy, reliability, and reproducibility. The accuracy of the detection data for each batch was ensured by including a standard curve and QC data in each analytical batch. These features examined in this study establish a solid foundation for the further use and statistical analysis of this method.

Although prior studies have described the method by which glycosylation end products are created, there has been no large-scale investigation comparing the results of CML and CEL in thousands of plasma samples to show an association with aging. The aim of this study was to simultaneously examine several of the factors that influence aging. Based on the data on the effects of aging on glucose metabolism, statistical analysis of different age groups showed that the trends of changes in these two indicators with aging were basically similar; specifically, they both showed a significant increase between 40 and 80 years, with an average increase of 9.06 and 8.00% per decade of aging, respectively.

However, there was a slight difference between genders: the percentage of increase in CML was 10.89 and 7.39% for males and females, respectively, with a difference of 3.5%; the percentage of increase in CEL was 8.56 and 7.76% for males and females, respectively, with a difference of 0.8%. The difference in age-related changes between genders was more significant in CML values than that of CEL, suggesting that clinically, CML is a more sensitive indicator for males, while for females, both CML and CEL are equally qualified as evaluation indicators.

In terms of the overall trend, both indicators showed a correlation trend with age in both male and female groups, and this correlation was similar in male and female samples. Hence, these two indicators can be recommended as assistant indicator with relatively broad applicability for assessing the impact of aging on glucose metabolism.

While CML and CEL have shown some utility as clinical indicators in studies related to aging, more research is needed to further validate their effectiveness and understand the underlying mechanisms. Recent research findings have suggested that CML is also influenced by the levels of acetaldehyde in healthy individuals [22]. There are also studies that have reported the impact of dietary sugars and related endogenous advanced glycation end products on the extent of chromosomal DNA damage in WIL2-NS cells [21]. These findings clearly indicate that aging is a complex process influenced by multiple factors, and using a single indicator can have only limited value. Therefore, a combination of multiple biomarkers and clinical indicators needs to be considered for a more accurate assessment of the risk due to aging and health status.

## Conclusion

In conclusion, the assay developed in this study fulfilled the clinical requirements for the rapid detection of CML and CEL in human plasma samples. Its sensitivity, precision, and accuracy were also qualified, and the results were accurate and reliable. The findings of this study provide information on the trends in changes of these two markers across different age groups with respect to the evaluation of glucose metabolism. However, further validation is required through additional research into the related mechanisms. The analytical detection method established in this study was accurate, reliable, and highly sensitive, providing a methodological and practical foundation for further research in the realm of human health monitoring.

## Data Availability

The datasets used and/or analysed during the current study available from the corresponding author on reasonable request. We declared that materials described in the manuscript, including all relevant raw data, will be freely available to any scientist wishing to use them for non-commercial purposes, without breaching participant confidentiality.
